# From Bits to Bedside: From Data to Knowledge in Vascular Surgery

**DOI:** 10.3390/jcm15187293

**Published:** 2026-09-19

**Authors:** Johannes Hatzl, Alexandru Barb, Jasmin Epple, Deborah De Basso, Wolfram Stein, Luna Zetsche, Martin Dugas, Dittmar Böckler

**Affiliations:** 1 Department of Vascular and Endovascular Surgery, University Hospital Heidelberg, 69120 Heidelberg, Germany; 2 Institute of Medical Informatics (IMI), University Hospital Heidelberg, 69120 Heidelberg, Germany

**Keywords:** vascular surgery, Data–Information–Knowledge, natural language processing, large language models

## Abstract

The Data–Information–Knowledge (DIK) hierarchy describes how clinical observations are progressively transformed into actionable knowledge. Although digital technologies are beginning to reshape vascular surgery, their impact is often discussed in isolation rather than as components of a continuous knowledge-generation process. This narrative review applies the DIK framework to examine how recent technological advances are transforming each stage of knowledge generation in vascular surgery. Emphasis was placed on data modeling, computer vision and natural language processing, as well as retrieval-augmented generation (RAG)-based large language model applications. Despite these advances, limitations remain, including data quality, external validation, generalizability, and the reliability and transparency of generated recommendations. Maximizing future knowledge generation requires coordinated advances across all three layers, especially on the data layer.

## 1. Introduction

**Figure 1:**
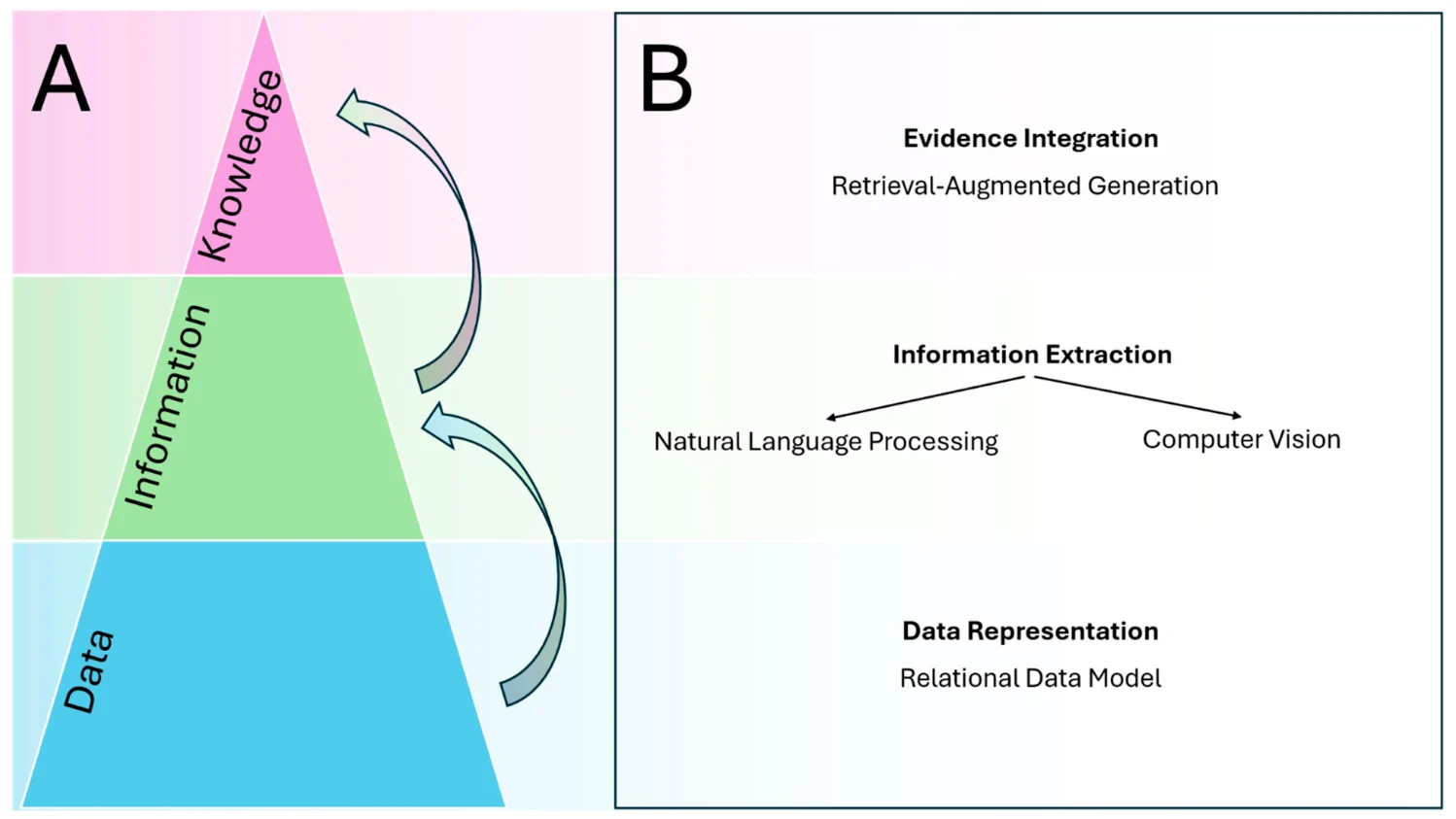
The Data–Information–Knowledge (DIK) hierarchy as a framework for knowledge generation in vascular surgery. (**A**) The DIK hierarchy illustrating the sequential transformation of clinical observations into actionable knowledge. (**B**) Contemporary technological advancements impact all layers of this pipeline: relational data models support data representation, computer vision and natural language processing enable information extraction, and retrieval-augmented generation facilitates evidence integration.

The Data–Information–Knowledge (DIK) hierarchy is a conceptual framework originating from information science that describes how observations are progressively transformed into actionable knowledge [1,2,3,4]. At its most fundamental level, data represents observations of reality. Through interpretation, these observations become information. Information can subsequently be integrated with existing scientific evidence and clinical experience to generate actionable knowledge. The DIK hierarchy is demonstrated in Figure 1A.

The DIK hierarchy provides a useful perspective on how clinical knowledge is generated. All three layers of this hierarchy are currently undergoing digital transformation in clinical medicine including vascular surgery. These technological advancements have often been reviewed independently from one another. However, considering them within a common conceptual framework highlights that they address different stages of the same knowledge-generating pipeline.

In this narrative review, we use the DIK hierarchy to discuss recent technological developments and their transformative impact on data collection, information, and knowledge generation in vascular surgery, emphasizing both their opportunities and limitations. 

## 2. Data

The data layer constitutes the digital representation of clinical reality. In vascular surgery, this includes—among others—patient history, physical examination, laboratory results, physiological measurements, imaging, procedural details, devices, and follow-up data. These raw observations represent the foundation upon which the information and knowledge layers are built upon.

Accordingly, the generation of information and knowledge depends on the completeness, accuracy, and reliability of underlying data. Regulatory and methodological frameworks, including for example “Good Clinical Data Management Practice” (GCDMP), the “Strengthening the Reporting of Observational Studies in Epidemiology” (STROBE) statement, or the “Reporting of Studies Conducted Using Observational Routinely collected Data” (RECORD) guidelines, emphasize standardized data collection, validation procedures, audit trails, transparent data processing, and comprehensive documentation [5,6,7]. Likewise, the European Artificial Intelligence Act explicitly identifies data quality as a prerequisite for trustworthy high-risk artificial intelligence systems [8].

Over the past decades, substantial effort has been devoted to harmonizing clinical datasets. Consensus reporting standards have addressed the issues of terminology, measurement methodology, outcome definitions, and scope of reporting, specifically for vascular surgery. These initiatives have considerably improved transparency, interoperability, and reproducibility [6,9,10,11].

However, most of these efforts predominantly address how clinical observations are termed, defined or communicated, and therefore operate primarily on the informational layer. Comparatively less attention has been directed towards the preceding question of how clinical reality itself should be represented within the data layer, as conceptualized within the DIK model.

Within the DIK model, the primary purpose of the data layer is not to provide interpretation but to preserve clinical observations with the lowest reasonably achievable level of abstraction. Interpretation belongs to the informational layer. Consequently, the data layer is supposed to maximize future analytical potential by preserving primary observations rather than condensing them within derived descriptors or classification systems wherever feasible. Strictly interpreted, for example for patient history taking, the actual conversation transcript between the healthcare provider and the patient belongs on the data layer, and the resulting interpretation and classification (e.g., “claudication”) already belong to the information layer.

This distinction becomes particularly apparent in vascular imaging. A computed tomography angiography examination may ultimately be represented—for example—by maximum aneurysm diameter, aneurysm neck length, aneurysm thrombus volume, iliac calcification score, or any anatomical classifications in a dataset. “Reporting standard” documents in vascular surgery are intended to ensure that these variables are measured and reported consistently. Nevertheless, every derived descriptor inevitably represents only a fraction of the information contained within the original imaging data file. The data layer constitutes the raw imaging file. Similar abstraction occurs throughout clinical data processing. Complex procedural details are condensed into variables such as technical success or target lesion revascularization. These abstractions seem indispensable for scientific reporting but inevitably discard information.

Recent developments have substantially increased the importance of preserving primary observations. Historically, clinically relevant information was extracted almost exclusively through human interpretation (and routed directly to the informational layer). Contemporary computational methods increasingly derive information directly from imaging, physiological signals, and free-text documentation. Consequently, primary data possess enduring scientific value because they remain amenable to repeated analysis as computational methods continue to evolve and are not yet foreseeable. In contrast, the informational layer remains largely fixed at the time it is curated and therefore reflects the analytical capabilities, definitions, and assumptions available at that point in time. Data discarded during abstraction into information can hardly be recovered.

Already on the data layer, substantial risks can be identified. The way the data is collected, archived, and used for subsequent steps can introduce bias in various ways. For specific tasks, such as analyzing computed tomography angiography for aortic morphometrics, usually dedicated models are being trained based on a sample dataset. If certain anatomies are underrepresented in that sample dataset, the software might underperform systematically on underrepresented subgroups, leading to bias that enters the subsequent layers. Therefore, additional efforts are necessary to establish accessible training data with the aim of reducing potential biases. 

Preserving clinical observations requires an appropriate computational representation of clinical reality. Unfortunately, clinical reality in vascular surgery is inherently complex. Patients frequently present with multiple comorbidities, repeated imaging studies, staged interventions, a vast variety of devices, complications, and lifelong surveillance. Representing these complex longitudinal treatment pathways represents a challenge in clinical database design.

Since its introduction by Edgar F. Codd in 1970, the relational database model has remained the predominant paradigm for structured clinical data management [12,13]. Rather than storing isolated variables, relational databases represent reality through entities, their attributes, and explicitly defined relationships. 

A fundamental principle of relational database design is normalization. Normalization organizes data to minimize redundancy, maintain consistency, and preserve relationships between observations by ensuring that each piece of data is represented only once according to its natural entity. Besides reducing storage redundancy, normalized data structures are easier to maintain and update and improve long-term data integrity. 

Building upon these principles, efforts focusing on the development of relational data models—or alternative data management systems complying with these requirements—for vascular surgery might facilitate the utilization of recently developed technologies by enabling accurate storage on the data layer. Such models aim to harmonize data representation across diagnoses, procedures, and institutions while preserving primary clinical observations within standardized computational structures. A prominent example is the Observational Medical Outcomes Partnership Common Data Model (OMOP CDM), which provides a standardized relational data structure and controlled vocabularies for representing longitudinal healthcare data across institutions. While originally developed to facilitate large-scale observational research, its emphasis on semantic interoperability and standardized data representation illustrates how computational data models can support the preservation and reuse of primary clinical observations [14,15]. Rather than replacing existing reporting standards, they complement them by extending harmonization from terminology and definition towards the representation of clinical reality itself with as little abstraction as reasonably achievable [16]. Implementing OMOP CDM in vascular surgery therefore requires mapping complex domain-specific data in an ontology: device characteristics (graft type, diameter, location), anatomical classifications (aneurysm morphology), procedural details (access routes, adjunctive procedures), and longitudinal imaging follow-up with standardized measurements. This implementation demands initial institutional investment but deems multicenter research otherwise impractical. Normalized structures allow automated surveillance systems to identify patients with concerning aneurysm growth patterns or detect device-related complications across centers—analyses requiring consistent data representation. As a result, when computational methods continue to evolve, clinical databases with the ability to accurately capture clinical reality might provide the most sustainable foundation and a valuable resource for future information extraction and knowledge generation. Importantly, any developed ontology needs to be reviewed for hidden bias potential and should operate on the data layer as much as reasonably achievable, since in medicine the data and information layers are easily confused.

Definitions of the terms “Data”, “Information”, and “Knowledge” in the context of vascular surgery are presented in Table 1.

## 3. Information

Within the DIK hierarchy, information arises through the interpretation and structuring of data into clinically meaningful descriptors. In vascular surgery, this traditionally includes—for example—anatomical measurements, morphological classifications, hemodynamic patterns, or procedural classifications. Unlike data, information already constitutes an abstraction of clinical reality, as selected aspects of the underlying observations are translated into classification systems or measurement methodologies suitable for scientific reporting.

Historically, information extraction has relied almost exclusively on clinician interpretation. Radiologists and vascular surgeons manually perform measurements, identify anatomical landmarks, classify disease according to established criteria, and derive structured information from imaging and clinical documentation. Considerable effort has been devoted to standardizing this process [14,15]. Nevertheless, clinically relevant interobserver variability remains a concern [17,18]. Even fundamental descriptors such as maximal aneurysm diameter or aneurysm neck morphology have demonstrated limited reproducibility [19,20]. Consequently, information generation has traditionally been labor-intensive, observer-dependent, and difficult to sustain.

Recent advances in artificial intelligence have the potential to fundamentally improve performance on the information layer. Within the DIK framework, contemporary tools for computer vision and natural language processing can be understood as information extraction applications, transforming data into information.

Automated aortic morphometry represents one of the most mature examples of this development. Modern software applications no longer perform isolated measurements but execute complete analytical workflows. Following image acquisition (data), convolutional neural networks automatically segment the aorta and branch vessels, generate vascular centerlines, identify anatomical landmarks, and derive comprehensive and extensive morphometric descriptions (information). Beyond maximum aneurysm diameter, contemporary systems routinely quantify neck morphology, vessel lengths, angulations, tortuosity, thrombus burden, calcification, lumen and aneurysm volumes. Consequently, information that previously required extensive human interpretation can now be translated automatically into usable information on which knowledge can be generated.

Several commercially available software applications have demonstrated that this approach is feasible. Applications such as PraevAorta 2 (Nurea, Bordeaux, France) and ARVA (Incepto, Paris, France) achieve automated morphometric measurements with agreement metrics comparable to experienced human observers while substantially reducing analysis time. More importantly, these systems do not simply reproduce manual workflows but enable comprehensive characterization of vascular anatomy at a scale that would be impractical through manual measurements alone. Large imaging repositories can therefore be analyzed reproducibly, facilitating multicenter morphometric studies, automated surveillance, and image-derived prediction models, largely eliminating human interpretation when translating data to information [21,22,23,24,25,26,27,28,29,30,31,32,33,34,35].

For example, Postiglione et al. performed one of the largest multicentre evaluations of automated aortic morphometry to date, including 350 CT angiograms from two institutions. Automated measurements demonstrated a median absolute error of 1.6 mm, which was essentially identical to the variability observed between human observers. In addition to maximum diameter measurements, the workflow simultaneously generated volumetric analyses of the entire aorta. By demonstrating expert-level performance under multicentre conditions, the study represented an important step from proof-of-concept development toward clinical validation of automated morphometric workflows [31].

Caradu et al. were among the first to demonstrate the feasibility of fully automated deep learning-based segmentation for abdominal aortic aneurysms. Using 101 preoperative CT angiograms, the authors compared automated segmentation and diameter measurements with expert-generated reference segmentations. Automated analysis achieved excellent agreement (Dice coefficient 0.95) and a mean absolute diameter difference of only 0.6 mm. In a subsequent study, the same group extended the methodology to postoperative EVAR surveillance, demonstrating that automated segmentation remained feasible despite the presence of endografts, although measurement errors increased to 2.3 mm. These studies established automated segmentation as a reliable basis for subsequent morphometric analysis [24,36].

The current transformation extends beyond vascular imaging. Natural language processing (NLP) enables the automated extraction of structured information from previously unstructured clinical documentation, including operative reports, discharge summaries, outpatient letters, multidisciplinary conference notes, and others. Data that was previously embedded within free text can thereby be transformed into usable information [37,38,39,40,41,42,43,44,45,46,47].

In vascular surgery, these applications appear to be just in their beginnings. An illustrative example of LLM-based information generation was presented by Flanagan et al., who investigated whether narrative operative reports could be automatically transformed into structured registry information. Using a HIPAA-compliant implementation of ChatGPT 3.5 Turbo (gpt-35-turbo) and ChatGPT 4.0 (gpt-4), the authors analyzed 150 vascular surgical operative reports and extracted variables required for the Vascular Quality Initiative (VQI) registry. Overall, 93% of registry fields were completed correctly, demonstrating that LLMs can accurately identify procedural details embedded within free-text documentation. Such applications have the potential to substantially reduce the manual effort associated with clinical documentation while improving the completeness and availability of structured data for quality assurance, registry-based research, and outcome analysis. Since operative reports are generated routinely during clinical care, this approach represents an efficient strategy for secondary use of existing clinical documentation without requiring changes to established clinical workflows [43].

Another approach was described by Kartsonis et al., who developed an NLP pipeline for automated identification and characterization of abdominal aortic aneurysms from free-text radiology reports. Across 3229 reports, the system achieved F1-scores between 96.7% and 99.4% for aneurysm detection and size extraction. Beyond demonstrating high technical accuracy, the study also highlighted a clinically relevant application of automated information extraction. By comparing NLP-derived findings with electronic health records, the authors identified patients with previously documented aneurysms who had not been enrolled in surveillance programs and facilitated follow-up in a subset of these patients. The study therefore illustrates that automated processing of routine radiological reports may not only generate structured data for research but also contribute directly to quality assurance and identification of patients requiring ongoing clinical surveillance [48].

Hamelink et al. investigated automated diameter measurements of the thoracic aorta using low-dose chest CT examinations. Mean absolute measurement differences were 1.3 mm compared with expert observers, with a segmentation failure rate of approximately 5%. The study demonstrated that automated morphometry can be applied beyond dedicated angiographic examinations and may enable opportunistic assessment of thoracic aortic disease using routinely acquired chest CT datasets [49].

Despite these advances, important limitations remain. Current applications continue to depend on training data with human annotation. Furthermore, complex anatomy, uncommon pathologies, and impaired image quality remain frequent causes of segmentation failure [27,50]. Likewise, external validation studies remain scarce, and generalizability across institutions, imaging protocols, scanners, and patient populations has not yet been comprehensively established. Similar challenges exist for natural language processing, where documentation styles, institutional terminology, and language-specific characteristics may substantially influence extraction performance and generalizability. Beyond these validation challenges, practical barriers limit clinical implementation. Most current AI systems require institutional resources: dedicated imaging infrastructure, high-performance computing, and personnel trained in both clinical medicine and informatics—investments many clinical centers cannot justify. And even when systems achieve strong performance in controlled settings, their effectiveness typically remains confined to the specific institutional context in which they were developed and tested. Variations in scanning protocols, scanner manufacturers, and software versions across institutions may degrade performance substantially. An AI system trained on one scanner type or imaging protocol may lose accuracy when applied to different equipment or institutional workflows. This institutional heterogeneity reflects real-world clinical practice but fundamentally challenges the development of universally applicable AI systems. Federated learning and transfer learning approaches are being explored to address these generalizability challenges, though their clinical effectiveness remains under investigation. Additionally, flawed outcomes may result if the informational and data layers are confused. For example, using training data originating from the informational layer, such as reimbursement databases or other databases of which the content is derived from previous interpretation and collected for various purposes, potential bias introduced via this interpretation or purpose could negatively impact a system’s performance in its given task. Nonetheless, recent developments in computer vision and NLP have the potential to transform the informational layer of the DIK hierarchy if carefully applied. As computational methods continue to mature, information generation is likely to become increasingly automated, more reproducible, and scalable, providing a high-quality foundation for subsequent knowledge generation (Figure 1B).

## 4. Knowledge

Knowledge emerges through the integration of information into clinical understanding and decision making. In contrast to information, knowledge contextualizes these observations by combining them with existing scientific evidence, clinical experience, and guideline recommendations.

Traditionally, this integrative process relied on the treating clinician. During routine care, vascular surgeons continuously synthesize heterogeneous sources of information, including imaging findings, physiological measurements, laboratory values, procedural details, previous medical history, and patient preferences. These observations are interpreted in the context of current evidence, clinical guidelines, and individual experience to arrive at diagnostic and therapeutic decisions. However, this integrative process is inherently limited by cognitive bandwidth, potential recall bias, and the challenge of synthesizing rapidly evolving evidence—constraints that become increasingly pronounced as medical knowledge expands.

Recent advances in RAG can fundamentally change the technological landscape of the knowledge layer [21,51,52]. The ability to process natural language enables synthetization of heterogeneous information sources, retrieval of relevant medical evidence, comparison of recommendations across clinical guidelines, summarization of scientific literature, and ultimately generation of context-specific suggestions. Within the DIK hierarchy, they can therefore be understood as knowledge integration systems (Figure 1B).

Rather than relying exclusively on the information encoded within model parameters of large language models (LLM), contemporary LLM systems increasingly retrieve external sources such as clinical guidelines or local databases during inference. This approach is particularly relevant in medicine, where evidence, guideline recommendations, and device-specific instructions evolve continuously. Consequently, recommendations can be explicitly grounded in current evidence while reducing dependence on static parametric knowledge. A RAG-based system does not generate new knowledge from new data; it integrates existing external evidence in context-specific responses. This suggests that it functions more as a knowledge integration layer than a data processing tool. Furthermore, retrieved sources can be linked directly to generated outputs, allowing clinicians to verify the underlying evidence and thereby improving transparency. This development shifts LLMs from general-purpose language models towards evidence-aware clinical assistants capable of supporting guideline-based decision making even in complex scenarios [53]. By bridging the gap between static model parameters and dynamic clinical evidence, RAG systems enable clinicians to maintain confidence in recommendations even as guidelines evolve and new evidence emerges—a critical requirement for sustainable clinical decision support.

Several applications have already demonstrated the practical value of this approach. The potential of RAG has recently been demonstrated in the field of vascular surgery through the development of VASC.AI, a specialty-specific chatbot combining a large language model with a curated vascular knowledge base comprising guidelines, landmark publications, and others. The system retrieves relevant evidence before generating a response, grounding recommendations in current vascular surgery literature [54]. Similar findings have been reported in other fields, such as radiology, where the RadioRAG framework combined LLMs with real-time retrieval and demonstrated improved answer accuracy as well as reduced hallucination rates compared with non-RAG baseline models.

In addition, multimodal AI enables processing of non-textual data, such as imaging, ECG, and others. The combination of multiple modes can improve discrimination. However, because errors at this layer can scale into diagnostic or therapeutic advice on an individual case-by-case level, in the future, these tools require thorough validation and, equally importantly, an educated “user” who is familiar with potentially non-obvious failure modes. Future studies will have to demonstrate the ability of any such system to ultimately improve outcomes.

Despite these advances, substantial limitations remain on the knowledge layer. Current LLMs have been shown to generate factually incorrect statements, omit relevant evidence, or produce recommendations that appear plausible despite lacking scientific support [55]. These risks are further amplified when systems are applied beyond their training domain or when external knowledge sources themselves contain outdated or conflicting recommendations. Their performance also depends heavily on prompt design, retrieval strategy, and the quality of external knowledge sources. Furthermore, reasoning processes remain only partially transparent, and responsibility for clinical decisions cannot be delegated to computational systems. Based upon these limitations, considerable harm could be caused at scale when widely adopted. Therefore, validation of those tools intended for diagnostic or therapeutic purposes is paramount prior to clinical use. Such validation should encompass not only technical accuracy but also assessment of clinical utility, workflow integration, impact on patient outcomes in real-world settings, and thorough analysis of potential bias on all three layers to identify biases propagating up to the knowledge layer.

Within the DIK hierarchy, this represents the final stage of digital transformation: after preserving clinical observations and automating information extraction, computational systems are increasingly beginning to support the integration of information into clinically actionable knowledge (Figure 1B).

## 5. Conclusions

Knowledge generation in vascular surgery can be understood as a continuous pipeline that transforms clinical observations (data) via interpretation (information) into actionable clinical decisions (knowledge). The DIK hierarchy provides a conceptual framework to follow this process. As discussed in this review, digital transformation is currently occurring simultaneously at every layer of the model, in clinical medicine and vascular surgery alike. The overall performance of this pipeline is likely a function of its weakest component. Importantly, advances in knowledge generation cannot compensate for incomplete or poorly represented information, just as increasingly sophisticated information extraction cannot recover data that was never captured. Only by addressing the current limitations on each layer can the full potential of recent digital advancements be utilized for the benefit of vascular surgical patients.

## Figures and Tables

**Table 1:** Definitions of the terms “Data”, “Information”, and “Knowledge” in the context of vascular surgery.

Term	Definition	Example
Data	Clinical observation with as little abstraction as reasonably achievable.	Computed tomography angiography imaging data (DICOM file), patient history transcript
Information	Quantified or structured descriptors extracted from the data layer.	Maximum infrarenal aortic diameter: 58 mm aneurysm; neck length: 3 mm
Knowledge	Interpretation and synthesis of information in the context of existing evidence and clinical understanding.	Anatomy is consistent with a juxtarenal abdominal aortic aneurysm, suggesting standard EVAR is unlikely to be suitable. Alternatives are suggested and prioritized considering guidelines and literature review

## Data Availability

No new data were created or analyzed in this study. Data sharing is not applicable to this article.

## Abbreviations

The following abbreviations are used in this manuscript:

DIK	Data–Information–Knowledge
GCDMP	Good Clinical Data Management Practice
STROBE	Strengthening the Reporting of Observational Studies in Epidemiology
RECORD	Reporting of Studies Conducted Using Observational Routinely collected Data
OMOP CDM	Observational Medical Outcomes Partnership Common Data Model
NLP	Natural language processing
RAG	Retrieval-augmented generation
LLM	Large language models
